# UpCoT: an integrated pipeline tool for clustering upstream DNA sequences of orthologous genes in prokaryotic genomes

**DOI:** 10.1007/s13205-016-0363-4

**Published:** 2016-02-16

**Authors:** P. V. Parvati Sai Arun, Jogadhenu S. S. Prakash

**Affiliations:** 1 Department of Plant Sciences, School of Life Sciences, University of Hyderabad, Hyderabad, 500046 India; 2 Department of Biotechnology and Bioinformatics, School of Life Sciences, University of Hyderabad, P. O. Central University, Hyderabad, 500046 India

**Keywords:** Clustering, Upstreams, Orthologs, Motif, Transcriptional units

## Abstract

UpCoT is a pipeline tool developed by automating the series of steps involved in prediction of *cis*-regulatory elements. UpCoT generates orthologs for each gene in target genome using bi-directional best blast hit against the reference genomes, then identifies potential orthologous transcriptional units using intergenic distance. Finally it generates the FASTA files containing upstream sequences of orthologous transcriptional units of each gene in target genome. The inputs of UpCoT are protein sequence files (*.faa), genome sequence files (*.fna) and gene co-ordinate files (*.ptt) for target and reference genomes. The clustered-upstream DNA sequences can be used by motif prediction tool, such as MEME, Bio-prospector, Gibbs motif sampler, MDscan for prediction of conserved DNA elements. We tested the performance of UpCoT by selecting the genome of *Synechocystis* sp PCC 6803 as the target and 13 different cyanobacterial genomes as reference. The clustered upstream sequences generated by UpCoT of *groES*, *ycf24* and *nirA* were used for *cis*-regulatory element prediction. The results were consistent with the experimentally identified *cis*-regulatory elements. Therefore, UpCoT is a reliable and automated pipeline package for prediction of orthologs, orthologous transcriptional units, and orthologous upstream sequences of a selected prokaryotic genome. UpCoT can be downloaded from http://jssplab.uohyd.ac.in/upcot/.

## Introduction

With the advent of fast and next generation automated DNA sequencing technologies, a number of microbial genomes have been sequenced during the past decade and the sequence information is available in various genome databases. Identification of *cis*-regulatory elements and the *trans*-acting factors of a sequenced genome is one of the major challenges to computational biologists for building a global gene regulatory network. Phylogenetic footprinting is one of the widely accepted computational method for predicting *cis*-regulatory elements for a given genome in question (Hardison 2000). This method can be considered as a two step process. The first step involves, identification of orthologs in the reference genomes, for each protein of a target genome by bidirectional best hit method, prediction of transcriptional units of target and reference genomes, generation of cluster of transcriptional units (CoTs), and finally clustering of upstream DNA sequences based on the generated CoT data (Wels et al. 2006). The second step involves scanning for conserved DNA elements in the clustered-upstream DNA sequences of a given CoT. Various computational tools, such as MEME, Bioprospector, Gibbs sampler, MDScan are used for predicting conserved DNA elements in a given set of DNA sequences and can be represented in the form of consensus pattern (Bailey and Elkan 1994; Liu et al. 2001, 2002; Mrazek 2009; Neuwald et al. 1995). There are many computational tools to perform the second step of phylogenetic foot printing but, they are not available to perform the first step. Further, the first step by itself is a multi-step process and requires lengthy computational procedure. On the other hand, the number of microbial genomes being sequenced is constantly increasing and demands for the development of an automated tool. Developing such a tool would facilitate the biologists to work easily on any microbial genome for quick generation of clustered-upstream DNA sequences for the target genome in question. Keeping the above facts in view, we developed an automated integrated pipeline called, UpCoT, which identifies the orthologs for proteins of target organism (tgCoGs), generates clusters of transcription units (tgCoTs), and cluster the upstream DNA sequences of tgCoTs. The output of the UpCoT can be directly used for prediction of *cis*-regulatory elements using any computational tool of user’s choice.

## Materials and methods

### Design of UpCoT interface

UpCoT web interface was designed using HTML, PHP and javascript to select and retrieve the genomes for analysis by UpCoT package. The *.faa, *.fna, and *.ptt files of 1840 prokaryotic genomes were downloaded from NCBI (ftp://ftp.ncbi.nlm.nih.gov/genomes/Bacteria) and incorporated in the web server, where UpCoT package has been maintained. User can select any of these genomes as target and reference files as input to UpCoT package. UpCoT package along with selected genomes for analysis can be downloaded from the web link, http://jssplab.uohyd.ac.in/upcot. The UpCoT package was developed using Perl programming language and is compatible for Windows and Linux operating systems.

### Description and accessibility of UpCoT web interface

**Fig. 1:**
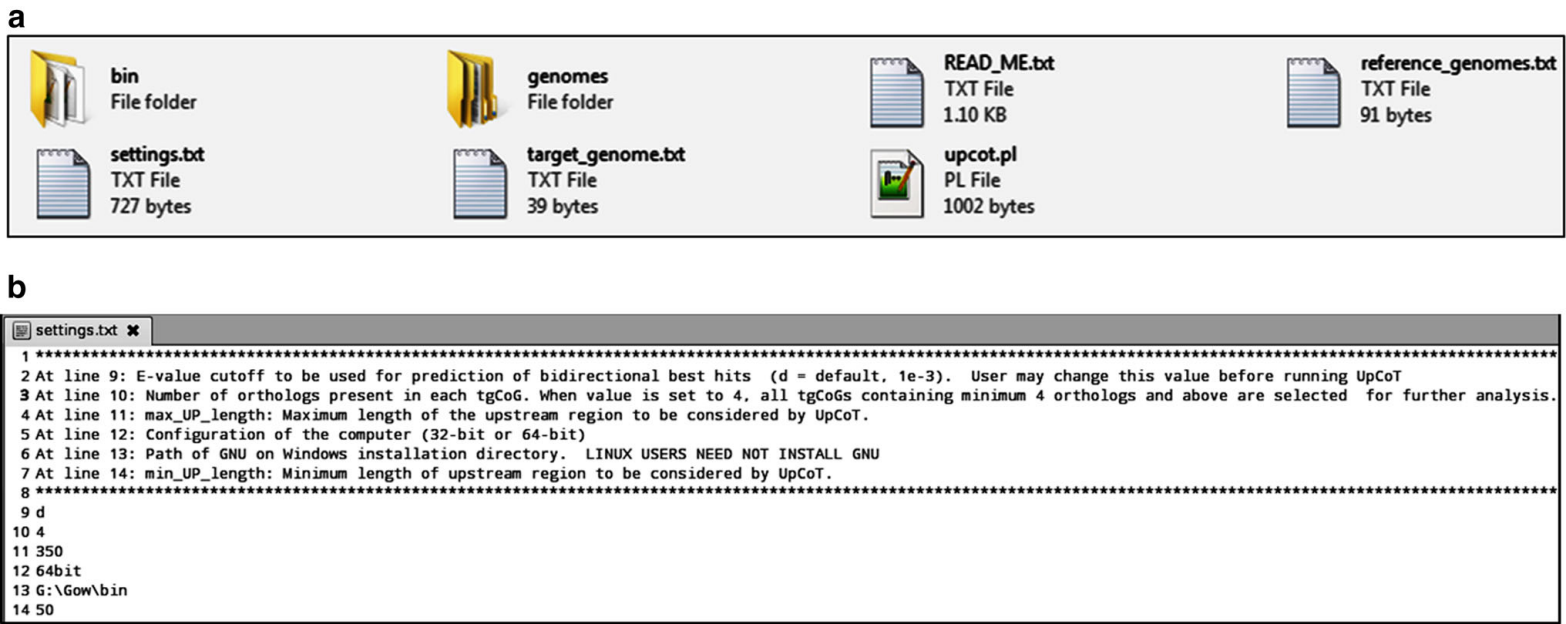
**a** Snapshot showing the directories, and files present in the UpCoT package. The “bin” directory contains Perl programs needed for running of UpCoT. The ‘genomes’ directory contains *.faa, *.fna, *.ptt files of selected target and reference genomes. ‘Read_Me.txt’ provides the instructions about how to use UpCoT package. The file ‘settings.txt’ provides the input parameters, as mentioned in **b**. The ‘upcot.pl’ is the main file which invokes all the Perl programs that are present in “bin” directory. **b** Snapshot showing the file contents of ‘settings.txt’. *E* value cutoff to be used for prediction of bidirectional best hits (*d* = default, 1 *e* ^−3^). User may change this value before running UpCoT. Number of orthologs present in each tgCoG. When value is set to 4, all tgCoGs containing minimum 4 orthologs and above are selected for further analysis. *Max_UP_length* maximum length of the upstream region to be considered by UpCoT. Configuration of the computer (32- or 64-bit). Path of GNU on Windows installation directory. *Min_UP_length* minimum length of upstream region to be considered by UpCoT

The web interface contains ‘Home’, ‘Help’ and ‘Contact’ links below the header. A brief introduction about the UpCoT is given in the homepage. A single click on ‘Select Genomes’ link navigates to a new web page displaying the list of prokaryotic genomes for selection as target genome. User can also select either windows or linux operating system, for downloading suitable executables to run the UpCoT package. Upon selecting the operating system and the target organism, a new page appears and prompts the user for selecting the reference genomes. Here the user can select any number of reference genomes for analysis and then click on the submit button. After the selection of both target and reference genomes, user can download the UpCoT package by a single click on ‘Download UpCoT’ tab. The UpCoT windows version needs a supporting software package GNU on windows to be installed, which is provided along with UpCoT package. UpCoT package also contains ‘settings.txt’, ‘README.txt’, ‘target_genome.txt’, and ‘reference_genomes.txt’ files (Fig. 1a). User may change the parameters, such as *E* value (default, *d* = 1 *e* ^−3^), orthologs count (default = 4), computer configuration (default = 64 bit), installation path of GNU on window (in case of windows user), minimum upstream length (min_UP length default = 50) and maximum upstream length (max_UP length default = 350 bp), in “settings.txt” file provided in the package (Fig. 1b). The “bin” directory provided in the UpCoT contains Perl programs developed for performing different tasks such as blastP, extraction of top scoring hits, prediction of bidirectional best hits, counting the number of orthologs, upstream sequence retrieval, and generating clustered-upstream sequences.

### UpCoT output

**Fig. 2:**
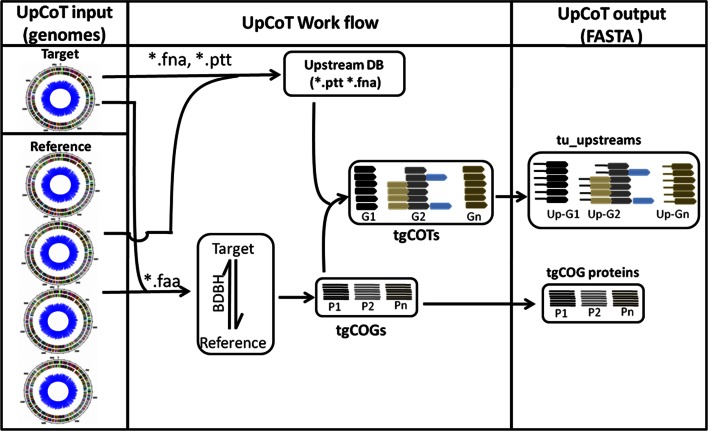
The schematic representation of the UpCoT input, UpCoT work flow and UpCoT output. The inputs for UpCoT are *.faa, *.fna, *.ptt files of target and reference genomes of user’s choice. UpCoT uses these files to generate tgCoGs by Bidirectional best hit method (BDBH) and the clusters of transcriptional units (tgCoTs). UpCoT groups the upstreams of each gene of a tgCoT to generate clustered-DNA upstreams of that tgCoT. All clustered-DNA upstreams of each tgCoT are saved into ‘tu-upstreams’ directory. Each output file is a text file named with ‘Up-ORF id’ of the target organism. UpCoT also generates the tgCoG protein sequences as text files. *G1* tgCoT of gene 1, *P1* tgCoG of gene 1, *Up-Gn* clustered-upstream sequences of gene ‘*n*’ of a target genome

UpCoT identifies the orthologs for each protein of target genome by bidirectional best hit method (BDBH) in the given reference genomes using BlastP (Altschul et al. 1990). After performing BDBH, UpCoT generates clusters of orthologs groups for target genome (tgCoGs) based on the orthologs count. For example, if the orthologs count is set to 4, tgCoGs containing four orthologs or above will be selected for further analysis. A directory named as “tgCoG_protein_sequences” is generated containing FASTA files of tgCoG protein sequences. In addition, UpCoT generates clusters of transcriptional units (tgCoTs) by reading the tgCoG files and also based on the length of their corresponding upstream DNA sequences. The minimum length of the upstream region (min_UP length default = 50) and the maximum length of the upstream region (max_UP length default = 350). It excludes the upstreams of the open reading frames, which are less than the defined nucleotide length. Reports suggest that the genes possessing an upstream region less than 40–50 bp are to be excluded from the computational prediction of *cis*-regulatory elements, we have set the minimum default integer value as 50 bp in the “settings.txt” file **(**Fig. 1b**)** (Conlan et al. 2005; Liu et al. 2008; Salgado et al. 2000). User may change the minimum length as per the requirement. When the actual length of the upstream region is greater than the minimum default length or user-defined minimum length, the program selects 350 bp upstream region of an ORF. When the upstream intergenic region is longer than 350 bp, the UpCoT considers only 350 bp upstream region, as the max_UP length is set to 350 bp. User may also change the max_length according to the requirement. Subsequently, UpCoT extracts and clusters the upstream DNA sequences of tgCoTs based on default or user defined integer values as upstream length given in “settings.txt” file. Upon completion of the whole process, a directory named “tu_upstreams” appears in the working directory of the UpCoT. This directory contain multiple text files, each with clustered-upstream DNA sequences of a tgCoT. Each text file is named with ORF number of the target gene. User can submit these upstream sequences for any motif prediction tool for identifying *cis*-regulatory elements. The entire work flow of UpCoT including inputs, the processes, and the outputs are depicted in (Fig. 2).

### Methodology used for testing UpCoT

We used the genome of *Synechocystis* sp. PCC 6803 (hereafter *Synechocystis*) as the target and *Acaryochloris marina* MBIC 11017, *Synechococcus* CC 9311, *Anabaena variabilis* ATCC 29413, *Synechococcus elongatus* PCC 6301, *Cyanothece* PCC 7424, *Synechococcus* JA 2 3B a 2 13, *Gloeobacter violaceus* PCC 7421, *Synechococcus* PCC 7002, *Microcystis aeruginosa* NIES 843, *Nostoc punctiforme* PCC 73102, *Thermosynechococcus elongatus* BP1, *Prochlorococcus marinus* MIT 9303, *Trichodesmium erythraeum* IMS 101 as reference genomes. We used the default *E* value (*d* = 1 *e* ^−3^), ortholog count as 4 and min_UP length as 50 and max_UP length as 500 bp for testing UpCoT. From the output generated by UpCoT, the text files with names *Slr2075*, *Slr0074* and *Slr0898* were selected from ‘tu_upstreams’ directory and submitted for the prediction of *cis*-regulatory elements using stand alone versions of MEME, Gibbs Motif Sampler, MDScan and Bioprospector.

## Results and discussion

### Performance analysis of UpCoT

The target genome, *Synechocystis* has 3172 open reading frames that code for proteins involved in various cellular processes and unknown proteins. Out of 3172 proteins, UpCoT has generated 2578 tgCoGs, each containing minimum four orthologs. This shows that 81 % of *Synechocystis* proteins are present in at least four selected cyanobacterial species. Orthologs identified by UpCoT for the selected proteins were retrieved from ‘tgCoG_protein_sequences’ directory and tested for accuracy. Table 1 shows the selected proteins and their orthologs along with their functional annotation. From (Table 1), it is clear that the orthologs identified by UpCoT for the proteins Slr2075 (GroES), Slr0074 (Ycf24), Slr0898 (NirA), Ssl2598 (PsbH), Smr0009 (PsbN), Sll0851 (PsbC) andSll0894 (PsbD) are accurate because their annotations are same as given in NCBI genome database.

**Table 1:** Orthologs identified by UpCoT for selected proteins of target organism, *Synechocystis* sp. PCC6803

*Synechocystis * sp. PCC 6803	Orthologous proteins identified for selected proteins of target organism, *Synechocystis* sp. PCC6803 by UpCoT						
	Slr2075 (GroES) co-chaperonin	Slr0074 (Ycf24) cysteine desulfurase activator complex subunit	Slr0898 (NirA) ferredoxin-nitrite reductase	Ssl2598 (PsbH) photosystem II reaction center protein H	Smr0009 (PsbN) photosystem II reaction center protein N	Sll0851 (PsbC) photosystem II CP43 protein	Sll0849 (PsbD) photosystem II D2 protein
*Acaryochloris marina* MBIC 11017	Am1_4412	Am1_1224	Am1_2984	Am1_1677	Am1_5511	Am1_1084	Am1_4084
*Anabaena variabilis* ATCC 29413	Ava_3627	Ava_0424	Ava_4539	Ava_2220	Ava_4451	Ava_1243	Ava_2512
*Cyanothece* PCC 7424	Pcc7424_1789	Pcc7424_4729	Pcc7424_1683	Pcc7424_1517	Pcc7424_4233	Pcc7424_0578	Pcc7424_2974
*Gloeobacter violaceus* PCC 7421	Gvip396	Gvip196	Gvip212	Gsl1716	Gvip411	Gvip319	Gvip318
*Microcystis aeruginosa* NIES 843	Mae_46070	Mae_23090	Mae_18410	Mae_11070	Mae_36550	Mae_41150	Mae_41160
*Nostoc punctiforme* PCC 73102	Npun r0830	Npun_f4822	Npun_r1528	Npun_f1088	Npun_r4314	Npun_r3636	Npun_f4553
*Prochlorococcus marinus* MIT 9303	P9303_05031	P9303_03021	P9303_29861	P9303_18181	P9303_24631	P9303_08421	P9303_08431
*Synechococcus* CC 9311	Sync_2283	Sync_2483	Sync_2898	Sync_1909	Syc_0309	Sync_0896	Sync_2586
*Synechococcus elongatus* PCC 6301	Syc1788_d	Syc2356_c	Syc0310_d	Syc0977_c	Syc1289_d	Syc0872_c	Syc0873_c
*Synechococcus* JA 2 3B a 2 13	Cyb_1619	Cyb_1405	Cyb_0034	Not identified	Cyb_1372	Cyb_0853	Cyb_1736
*Synechococcus* PCC 7002	Synpcc7002_a2457	Synpcc7002_a1814	Synpcc7002_a1827	Not identified	Synpcc7002_a0809	Synpcc7002_a1559	Synpcc7002_a2199
*Thermosynechococcus elongatus* BP1	Tll0186	Tll0490	Tlr1349	Tsr0149	Tsr1387	Tlr1631	Tlr1630
*Trichodesmium erythraeum* IMS 101	Tery_4326	Tery_4355	Tery_1068	Not identified	Tery_2867	Tery_0513	Tery_1230

*Synechocystis* was used as target and other selected cyanobacterial species were used as reference organisms. Functional annotation is given in parenthesis and is based on NCBI genome database (ftp://ftp.ncbi.nlm.nih.gov/genomes/Bacteria)

### Analysis of clustered-upstream DNA sequences for selected tgCoTs

UpCoT has generated 2578 text files each containing clustered-upstream DNA sequences of a tgCoT. Out of which, the clustered upstream DNA sequences of *slr2075*, *slr0074* and *slr0898* were submitted for *cis*-regulatory element prediction, as the regulatory elements for these genes were previously experimentally demonstrated to be the target sites for known transcription factors. The clustered-upstreams were submitted to four different motif prediction tools as described in the materials and methods. (Table 2) shows the predicted *cis*-regulatory elements which were identified in the clustered-upstreams of the above selected tgCoTs. In *Synechocystis* the gene *slr2075* encodes for co-chaperonin GroES. HrcA, a transcriptional repressor has been reported regulate the expression of the *groESL* operon by binding to a 9-bp inverted repeat *TTAGCACTC [N9] GAGTGCTAA* (Nakamoto et al. 2003; Zuber and Schumann 1994). When the clustered-upstream sequences of *slr2075*-tgCoT was submitted as input to motif prediction tools the same inverted repeat was predicted by MEME, Gibbs motif sampler and Bioprospector (Table 2). The *SufR* is a negative transcriptional regulator of *sufBCDS* operon in *Synechocystis*. *SufR* binds to *cis*-acting element, *CAAC*-*N6*-*GTTG* located between the divergently transcribed *sufR* gene and the *sufBCDS* operon, and acts as a repressor of the *sufBCDS* operon and as an auto-regulator of its own gene, *sufR* (Wang et al. 2004). Motif prediction tools MEME, MDScan and Bioprospector generated the same element upon submission of clustered-upstream sequences of *slr0074*-tgCoT (Table 2). A number of nitrogen assimilation genes are regulated by the global transcriptional regulator NtcA, that acts as both an activator and repressor (Aichi et al. 2001). The binding site of NtcA is reported to be a tri-nucleotide inverted repeat *GTA N*(*8*) *TAC.* The ORF, *slr0898* codes for Ferredoxin-nitrite reductase (NirA) in *Synechocystis*. Bioprospector tool has predicted the NtcA binding site in the clustered-upstream DNA sequences of tgCoT-*slr0898* (Table 2). Thus, based on the identification of experimentally validated *cis*-regulatory elements for clustered upstreams oftgCoTs, we suggest that UpCoT is suitable for extracting and clustering of upstreams for any group of microbial genomes with accuracy and can be used for phylogenetic foot printing, promoter prediction, sRNA mapping and TSS prediction.

**Table 2:**
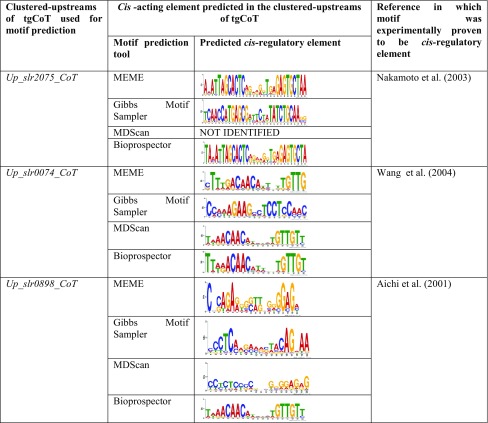
*cis*-regulatory elements identified in the clustered-upstreams of selected tgCoTs generated by UpCoT

The clustered-upstreams of slr2075-tgCoT (Up_slr2075_CoT), slr0074-tgCoT (Up_slr0074_CoT) and slr0898-tgCoT (Up_slr0898_CoT) were submitted to MEME, Gibbs Motif Sampler, MDScan and Bioprospector tools for identifying *cis*-regulatory elements. The predicted *cis*-regulatory elements are shown as a consensus sequence. The predicted conserved sequences were consistent with the previously published and experimentally validated *cis*-regulatory elements

## Conclusion

UpCoT is an automated software that can perform prediction of bidirectional best hits, clusters of transcriptional units (tgCoTs) and grouping of upstream DNA sequences for the predicted tgCoTs in a single step. It can be used as a tool by biologists to work on available microbial genomes for prediction of *cis*-regulatory elements using phylogenetic foot printing. UpCoT can be downloaded from http://jssplab.uohyd.ac.in/upcot/.

## References

[CR1] Aichi M, Takatani N, Omata T (2001). Role of NtcB in activation of nitrate assimilation genes in the cyanobacterium *Synechocystis* sp. strain PCC 6803. J Bacteriol.

[CR2] Altschul SF, Gish W, Miller W, Myers EW, Lipman DJ (1990). Basic local alignment search tool. J Mol Biol.

[CR3] Bailey TL, Elkan C (1994) Fitting a mixture model by expectation maximization to discover motifs in biopolymers. In: Proceedings/international conference on intelligent systems for molecular biology; ISMB international conference on intelligent systems for molecular biology, vol 2, pp 28–36 7584402

[CR4] Conlan S, Lawrence C, McCue LA (2005). *Rhodopseudomonas palustris* regulons detected by cross-species analysis of alphaproteobacterial genomes. Appl Environ Microbiol.

[CR5] Hardison RC (2000). Conserved noncoding sequences are reliable guides to regulatory elements. Trends Genet TIG.

[CR6] Liu X, Brutlag DL, Liu JS (2001) BioProspector: discovering conserved DNA motifs in upstream regulatory regions of co-expressed genes. In: Pacific symposium on biocomputing, pp 127–138 11262934

[CR7] Liu XS, Brutlag DL, Liu JS (2002). An algorithm for finding protein-DNA binding sites with applications to chromatin-immunoprecipitation microarray experiments. Nat Biotechnol.

[CR8] Liu J, Xu X, Stormo GD (2008). The *cis*-regulatory map of *Shewanella* genomes. Nucleic Acids Res.

[CR9] Mrazek J (2009). Finding sequence motifs in prokaryotic genomes—a brief practical guide for a microbiologist. Brief Bioinf.

[CR10] Nakamoto H, Suzuki M, Kojima K (2003). Targeted inactivation of the hrcA repressor gene in cyanobacteria. FEBS Lett.

[CR11] Neuwald AF, Liu JS, Lawrence CE (1995). Gibbs motif sampling: detection of bacterial outer membrane protein repeats. Protein Sci Publ Protein Soc.

[CR12] Salgado H, Moreno-Hagelsieb G, Smith TF, Collado-Vides J (2000). Operons in *Escherichia coli*: genomic analyses and predictions. Proc Natl Acad Sci USA.

[CR13] Wang T, Shen G, Balasubramanian R, McIntosh L, Bryant DA, Golbeck JH (2004). The sufR gene (sll0088 in *Synechocystis* sp. strain PCC 6803) functions as a repressor of the sufBCDS operon in iron-sulfur cluster biogenesis in cyanobacteria. J Bacteriol.

[CR14] Wels M, Francke C, Kerkhoven R, Kleerebezem M, Siezen RJ (2006). Predicting *cis*-acting elements of *Lactobacillus plantarum* by comparative genomics with different taxonomic subgroups. Nucleic Acids Res.

[CR15] Zuber U, Schumann W (1994). CIRCE, a novel heat shock element involved in regulation of heat shock operon dnaK of *Bacillus subtilis*. J Bacteriol.

